# Regulator of G protein signalling 14 attenuates cardiac remodelling through the MEK–ERK1/2 signalling pathway

**DOI:** 10.1007/s00395-016-0566-1

**Published:** 2016-06-13

**Authors:** Ying Li, Xiao-hong Tang, Xiao-hui Li, Hai-jiang Dai, Ru-jia Miao, Jing-jing Cai, Zhi-jun Huang, Alex F. Chen, Xiao-wei Xing, Yao Lu, Hong Yuan

**Affiliations:** Center of Clinical Pharmacology, The Third Xiangya Hospital, Central South University, 138 Tong-Zi-Po Road, Changsha, 410013 Hunan People’s Republic of China; Department of Cardiology, The Third Xiangya Hospital, Central South University, 138 Tong-Zi-Po Road, Changsha, 410013 Hunan People’s Republic of China; Department of Pharmacology, School of Pharmaceutical Sciences, Central South University, Changsha, 410078 Hunan People’s Republic of China; Center for Experimental Medicine Research, The Third Xiangya Hospital, Central South University, Changsha, 410013 Hunan People’s Republic of China

**Keywords:** Cardiac remodelling, Cardiac dysfunction, RGS14, MEK1/2, ERK1/2

## Abstract

In the past 10 years, several publications have highlighted the role of the regulator of G protein signalling (RGS) family in multiple diseases, including cardiovascular diseases. As one of the multifunctional family members, RGS14 is involved in various biological processes, such as synaptic plasticity, cell division, and phagocytosis. However, the role of RGS14 in cardiovascular diseases remains unclear. In the present study, we used a genetic approach to examine the role of RGS14 in pathological cardiac remodelling in vivo and in vitro. We observed that RGS14 was down-regulated in human failing hearts, murine hypertrophic hearts, and isolated hypertrophic cardiomyocytes. Moreover, the extent of aortic banding-induced cardiac hypertrophy and fibrosis was exacerbated in *RGS14* knockout mice, whereas *RGS14* transgenic mice exhibited a significantly alleviated response to pressure overload. Furthermore, research of the underlying mechanism revealed that the RGS14-dependent rescue of cardiac remodelling was attributed to the abrogation of mitogen-activated protein kinase (MEK)–extracellular signal-regulated protein kinase (ERK) 1/2 signalling. The results showed that constitutive activation of MEK1 nullified the cardiac protection in *RGS14* transgenic mice, and inhibition of MEK–ERK1/2 by U0126 reversed *RGS14* deletion-related hypertrophic aggravation. These results demonstrated that RGS14 attenuated the development of cardiac remodelling through MEK–ERK1/2 signalling. RGS14 exhibited great potential as a target for the treatment of pathological cardiac remodelling.

**Electronic supplementary material:**

The online version of this article (doi:10.1007/s00395-016-0566-1) contains supplementary material, which is available to authorized users.

## Introduction

Heart failure is the end stage of almost all cardiac diseases, resulting in increased morbidity and mortality. Cardiac remodelling, including hypertrophy and fibrosis, is the major independent risk factor for heart failure, which usually develops in response to hypertension, myocardial infarction, valvular heart disease, and endocrine disorders [6, 26, 44]. In recent decades, distinct signal transduction pathways have been identified to be involved in the development of cardiac remodelling [12, 15, 23, 24]. Among the most prominent signal transducers are the mitogen-activated protein kinase (MAPK), calmodulin-dependent phosphatase, and JAK–STAT signalling pathways [3, 11, 18], which are largely associated with G protein-coupled receptor (GPCR)-mediated signalling [56].

GPCRs constitute a large family of receptors that sense molecules outside the cell and activate intracellular signal transduction pathways. These receptors have been widely implicated in the cardiovascular system [54]. Perturbations in GPCR signalling could lead to pathological changes and contribute to various cardiovascular diseases, including hypertension, arrhythmia, and myocardial ischemia. Nearly one-third of the current pharmaceuticals on the market targeting GPCRs, such as angiotensin II receptor blockers, β-adrenergic receptor blockers, and luteinizing hormone-releasing hormone agonists, have had great success in treating human diseases [7, 32, 33]. Therefore, a better understanding of the modulatory mechanism of GPCRs in hypertrophic hearts might have great significance for improving the treatment of cardiac hypertrophy and heart failure.

Regulators of G protein signalling (RGS) were originally identified for their ability to accelerate the activity of Gα GTPase, which could reduce the amplitude and duration of GPCR effects. On the basis of target specificity, protein stability, and subcellular localization, the RGS protein superfamily is divided into four subfamilies: R4/B, R7/C, R12/D, and R2/A [13]. To date, at least 20 RGS proteins have been identified in cardiomyocytes and fibroblasts [25, 43, 51, 64, 68]. Previous studies demonstrated that several types of RGS proteins are involved in multiple pathophysiological processes in the heart, such as arrhythmia, heart failure, and hypertension [19, 46, 49, 50, 65, 66]. For example, Klaiber et al. demonstrated that RGS2 was involved in the anti-hypertrophic effects of cardiac atrial natriuretic peptide (ANP) [28].

RGS14, belonging to the R12/D subfamily, is a complex with multi-domain structures. Differing from other RGS, RGS14 contains two G-interacting domains: the RGS domain and the carboxyl terminal GoLoco domain. In addition, a tandem of two *Ras*-binding domains with affinity for the small GTPases *Ras* and *Rap* is located between the RGS and GoLoco domains [57, 58, 69]. It has been reported that RGS14 plays essential roles in cellular mitosis [8, 40, 41], birth process promotion [29], and phagocytosis by activating αMβ2 integrin [34]. Studies also revealed a role for RGS14 in suppressing synaptic plasticity in hippocampal CA2 neurons by integrating G protein and the MAPK signalling pathway [30, 61]. However, the exact role of RGS14 in the heart, particularly in response to stress stimuli, has not been investigated, although the expression of RGS14 in heart tissues has been confirmed by many studies [25, 55, 68]. Therefore, it is attractive and meaningful to determine the role and the underlying mechanism of RGS14 in pathological cardiac remodelling. In the present study, we explored if RGS14 expression was altered in hypertrophic hearts and further investigated the crucial role of RGS14 in cardiac remodelling by gain-of-function and loss-of-function approaches. The potential downstream mechanism of RGS14 in cardiac remodelling was well investigated.

## Methods and materials

### Reagents

Foetal calf serum (FCS) was obtained from HyClone (Shanghai, China). The antibodies and their commercial sources are listed below: Cell Signaling Technology (Beverly, MA): U0126 (#9903), anti-mitogen-activated protein kinase 1/2 (MEK1/2) (#9122), anti-phospho-MEK1/2 (#9154), anti-extracellular signal-regulated protein kinase 1/2 (ERK1/2) (#4695), anti-phospho-ERK1/2 (#4370), anti-c-Jun N-terminal kinase 1/2 (JNK1/2) (#9258), anti-phospho-JNK1/2 (#4668), anti-p38 (#9212), and anti-phospho-p38 (#4511); Santa Cruz Biotechnology, Inc.: anti-ANP (#sc20158) and anti-β-myosin heavy chain (β-MHC) (#sc53090); Aviva Systems Biology: anti-RGS14 (#OAAF04168); and Bioworld Technology: anti-GAPDH (#MB001). The bicinchoninic acid (BCA) protein assay kit was obtained from Pierce (Rockford, IL, USA). All other reagents, including the cell culture reagents, were purchased from Sigma.

### Source of human hearts

The failing human heart samples were obtained from the left ventricle (LV) of dilated cardiomyopathy (DCM) patients after heart transplantation. The control samples were collected from the LV of normal heart donors who died because of an accident. The Institutional Review Board (IRB) of the Third Xiangya Hospital, Central South University approved the study. The relatives of the heart donors signed informed consent.

### Mice

The Animal Care and Use Committee affiliated with the IRB of the Third Xiangya Hospital, Central South University approved all animal experimental protocols. All animals were housed in a light—(12 h light/12 h dark), temperature-controlled environment, and humidity-controlled environment. Food and water were available ad libitum. The animal models used in this study are described below.

### Cardiac-specific *RGS14*-overexpressing mice

Full-length mouse *RGS14* Complementary DNA (cDNA) (OriGene, MC204443) was ligated into the chicken β-actin gene (CAG) promoter expression vector, which was linearized and purified using the QIAquick Gel Extraction Kit (Qiagen, 28704). This DNA construct was microinjected into fertilized mouse embryos (C57BL/6J background). Founder transgenic mice were identified by tail DNA amplification and then bred with C57BL/6J mice. Tail genomic DNA was identified using polymerase chain reaction (PCR). The following primers were used for the PCR amplification of the CAG gene promoter: forward, 5′-CCCCCTGAACCTGAAACATA-3′; reverse, 5′-CTGCGCTGAATTCCTTCTTC-3′. The expected size for the amplification product was 579 bp. The *RGS14* flox mice were crossed with *α*-*MHC*-*MerCreMer* transgenic mice (Jackson Laboratory, 005650) to generate cardiac-specific *RGS14*-TG mice. Four independent transgenic lines were established. To induce RGS14 expression specifically in the heart, 6-week-old double transgenic mice were injected intraperitoneally with tamoxifen (80 mg/kg per day; Sigma, T-5648) for 5 consecutive days to cause Cre-mediated CAT gene excision. *CAG*-*CAT*-*RGS14/MHC*-*Cre* mice without tamoxifen administration (CRMC) served as the control group.

### Generation of *RGS14* knockout mice

Directive sequences of the target site for the *RGS14* gene in the mouse were predicted by the online CRISPR design system (http://crispr.mit.edu) (Fig. 3a). A pair of oligomers (oligo1, TAGGGGCCTGGGAACCTGCAGTGC; oligo2, AAACGCACTGCAGGTTCCCAGGCC) was cloned into the BsaI restriction site of the pUC57-single guide RNA (sgRNA) expression vector (Addgene, 51132). DNA was amplified by PCR with primers spanning the T7 promoter and sgRNA regions (forward primer, GATCCCTAATACGACTCACTATAG; reverse primer, AAAAAAAGCACCGACTCGGT). The sgRNA was transcribed by the MEGAshortscript kit (Ambion, AM1354) and purified by the miRNeasy Micro kit (Qiagen, 217084). The Cas9 expression plasmid (Addgene 44758) was linearized with PmeI and used as the template for in vitro transcription using the T7 Ultra Kit (Ambion, AM1345). Cas9 and sgRNA mRNA injections of single-cell embryos were performed by the FemtoJet 5247 microinjection system. Genomic DNA was extracted, and a 405 bp DNA fragment overlapping the sgRNA target site was amplified by PCR with the following primers: *RGS14*-F, 5′-CTGTGTGGACACTCCCATCC-3′; and *RGS14*-R, 5′-ACCACAGAGAGAAGCAGCAC-3′. The purified PCR product was denatured and reannealed in NEB Buffer 2 to form heteroduplex DNA that was digested with T7EN (NEB, M0302L) for 45 min and analyzed by 3.0 % agarose gel (Fig. 3b). These mice were sequenced to select for frameshift mutations (Fig. 3c). The following primers were used to screen F1 and F2 offspring: *RGS14*-F, 5′-CTGTGTGGACACTCCCATCC-3′; and *RGS14*-R, 5′-ACCACAGAGAGAAGCAGCAC-3′. Finally, *RGS14* knockout (*RGS14*-KO or *RGS14*^−/−^) mice were generated and identified as shown in Fig. 3d, e. Littermate controls of the *RGS14*-KO mice were wild-type mice (WT or *RGS14*^+/+^).

### Cardiac-specific *CaMEK1*-TG and *CaMEK1/RGS14* double TG mice

To obtain *CaMEK1* flox mice, the coding sequence of mouse *MEK1* S218D/S222D cDNA was ligated into the CAG promoter expression vector. This DNA construct was microinjected into fertilized mouse embryos (C57BL/6J background), and the resulting TG mice were PCR-genotyped using tail genomic DNA and the following primers: forward, 5′-CCCCCTGAACCTGAAACATA-3′; and reverse, 5′-CTGCGCTGAATTCCTTCTTC-3′. The expected size for the amplification product was 515 bp. The *CaMEK1* flox mice were crossed with *α*-*MHC*-*MerCreMer* transgenic mice, which were obtained from the Jackson Laboratory (No. 005650), to generate *CAG*-*MEK1/MHC*-*Cre* mice. Tamoxifen (80 mg/kg per day; Sigma, T-5648) was then injected into the *CAG*-*MEK1/MHC*-*Cre* mice containing the *CaMEK1* gene at 6 weeks of age for 5 consecutive days. *CAG*-*MEK1/MHC*-*Cre* mice without tamoxifen administration (CMMC) served as the control group. Finally, the CRMC mice were crossed with the CMMC mice and treated with tamoxifen to generate *CaMEK1/RGS14* double transgenic (DTG) mice.

### Aortic banding surgery

Aortic banding (AB) was performed as described previously [22, 37]. Briefly, mice were anesthetized using an intraperitoneal injection of sodium pentobarbital (50 mg/kg, Sigma) and ventilated with room air using a small animal ventilator (model VFA-23-BV, Kent Scientific, USA). The mice were kept warm on a heating pad until they regained consciousness. The left chest was opened after blunt dissection at the second intercostal space, and the thoracic aorta was identified. We tied the thoracic aorta to a 27 G or 26 G needle with a 7-0 silk suture depending on the body weight. The needle was removed quickly after the ligation, and the thoracic cavity was closed. Finally, the adequacy of aortic constriction was determined by the Doppler analysis. The mice in the control group were subjected to the same procedure without ligation of the aorta.

### Echocardiography evaluation

After the indicated times, the surviving mice were anesthetized using 1.5–2 % isoflurane and then subjected to echocardiography to examine cardiac function and structure, as previously described [21]. Briefly, a Mylab30CV ultrasound system switched to M-mode tracings with a 15 MHz probe was used to determine echocardiography. The LV end-diastolic dimension (LVEDd), LV end-systolic dimension (LVESd), and LV fractional shortening [FS (%) = (LVEDd-LVESd)/LVEDd × 100 %] were measured from the short axis of the LV at the level of the papillary muscles.

### Histological analysis and immunofluorescence staining

The animals were sacrificed 4–8 weeks after the AB or sham surgery. The hearts were harvested, arrested in diastole with 10 % potassium chloride solution, fixed with 10 % formalin, dehydrated, and embedded in paraffin. Paraffin-embedded hearts were cut transversely into 4–5 μm sections. Sections at the mid-papillary muscle level were stained with hematoxylin and eosin (H&E) and picrosirius red (PSR) to calculate the cardiomyocyte cross-sectional area (CSA) and collagen deposition volume, respectively. Fluorescein isothiocyanate-conjugated wheat germ agglutinin (WGA) was used to visualize the size of the cardiomyocytes. The immunofluorescence analysis was performed using the standard immunocytochemical techniques. Cardiomyocyte CSA, interstitial collagen deposition and perivascular collagen deposition were measured using the Image-Pro Plus 6.0 software.

### Quantitative real-time PCR and western blotting

Total mRNA was isolated from heart tissues or neonatal rat cardiomyocytes (NRCMs) using TRIzol reagent (Invitrogen). cDNA, which was obtained by reverse transcription of RNA, was synthesized using the Transcriptor First Strand cDNA Synthesis kit (Roche). Quantitative real-time PCR was performed using SYBR Green (Roche), and the relative expression of the target genes was calculated. GAPDH was measured and used for normalization. Cardiac tissue and cultured cardiomyocytes were lysed in RIPA lysis buffer, and the protein concentration was determined with a BCA protein assay kit. The proteins (50 μg) were resolved via SDS-PAGE (Invitrogen) and transferred to a PVDF membrane (Millipore), which was then subsequently blocked with milk. After overnight incubation with the indicated primary antibodies at 4 °C, the membranes were washed at least three times and then incubated with a secondary antibody for 1 h at room temperature. Finally, enhanced chemiluminescence-treated membranes were visualized using a FluorChem E imager (ProteinSimple, FluorChem E). The results were normalized to GAPDH.

### Cardiomyocyte and cardiac fibroblast culture and infection with recombinant adenoviral vectors

The heart ventricles of 1- to 2-day-old Sprague–Dawley rats were enzymatically dissociated into individual cardiomyocytes in PBS containing 0.03 % trypsin and 0.04 % type II collagenase. Fibroblasts were then removed by a differential attachment technique, and the NRCMs were plated at a density of 1 × 10^6^ cells/well in six-well plates and cultivated in DMEM/F12 medium containing 20 % FCS, penicillin/streptomycin, and bromodeoxyuridine to inhibit fibroblast proliferation. The cardiomyocytes were maintained in serum-free DMEM/F12 for 12 h and then treated with angiotensin II (Ang II, 1 μM) for 24 or 48 h to induce hypertrophy.

To obtain cardiac fibroblasts, the adherent non-myocyte fractions obtained during pre-plating were grown in DMEM containing 10 % FCS to confluence and passaged with trypsin-EDTA. All experiments were performed on cells from the first or second passages. Cardiac fibroblasts were placed in DMEM medium containing 0.1 % FCS for 24 h before the stimulation by Ang II for 24 h, and the expression of RGS14 was investigated.

Finally, cardiomyocytes were infected with adenoviral RGS14 (AdRGS14) to overexpress RGS14, and an adenoviral vector encoding the green fluorescent protein gene (AdGFP) was infected into cardiomyocytes as a control group. Adenoviral short hairpin RGS14 (AdshRGS14) constructs were obtained and infected into cardiomyocytes to knockdown RGS14 expression, and an adenoviral short hairpin RNA (AdshRNA) was used as the non-targeting control. NRCMs were infected with different adenoviruses in diluted medium for 12 h.

### Treatment of mice with U0126

U0126, an inhibitor of MEK1/2, was dissolved in dimethyl sulfoxide (DMSO) at a volume of 1 ml per 100 g of body weight and it was injected intraperitoneally into mice every 3 days (1 mg/kg) after AB. The control group was injected with a similar volume of DMSO.

### Statistical analysis

The results are expressed as the mean ± standard deviation (SD). All data were analyzed using Student’s two-tailed *t* test and analysis of variance (ANOVA) to compare the means of two groups of samples and multiple groups, respectively. The * T* approximation test was used for the analysis when the sample was less than 7. All statistical analyses were performed with the SPSS software (version 17.0). *P* < 0.05 was considered statistically significant.

## Results

### RGS14 expression is decreased in hypertrophic hearts

**Fig. 1:**
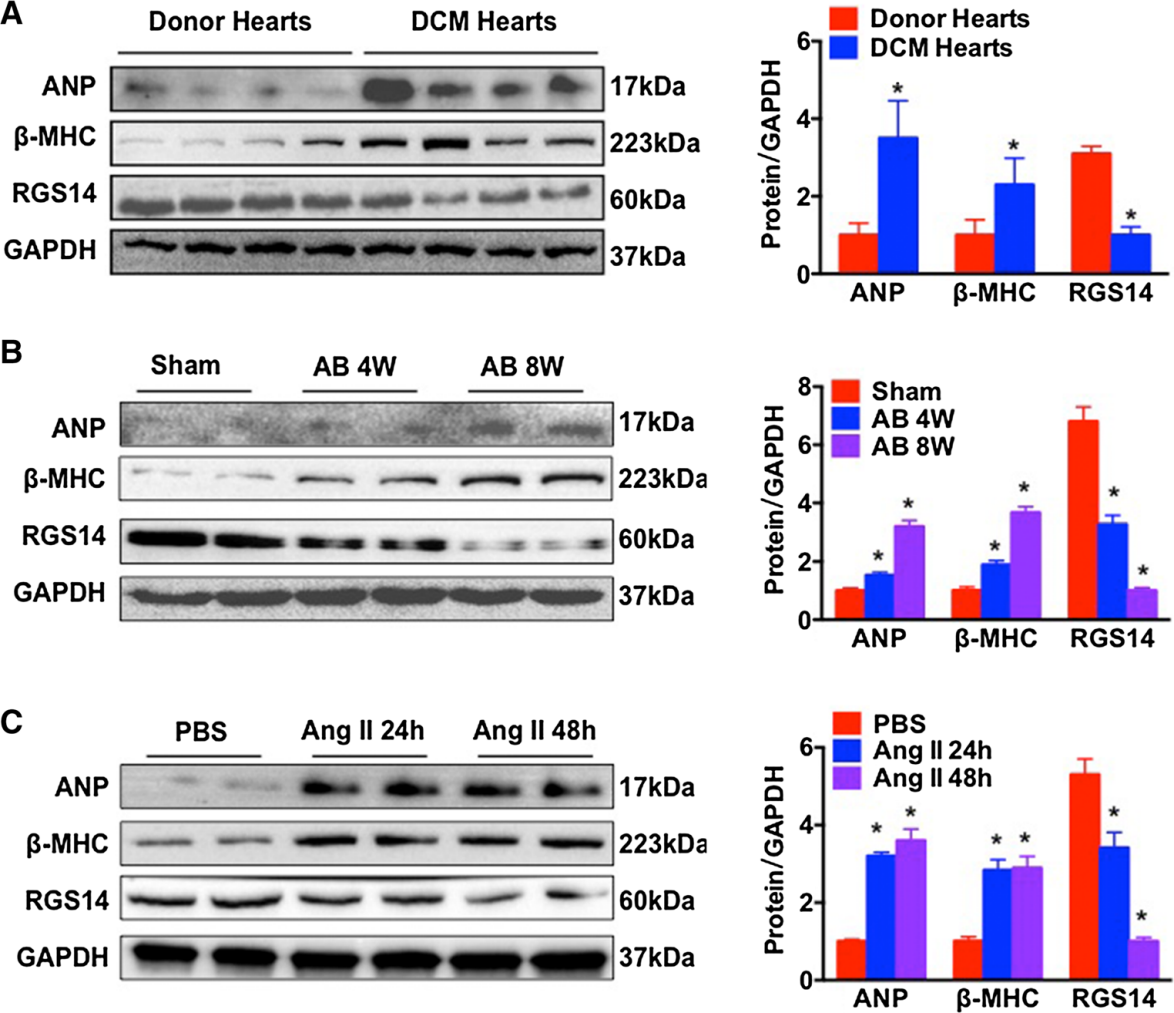
RGS14 is down-regulated in the failing heart and in the experimental hypertrophic models. **a** Western blot analysis of ANP, β-MHC, and RGS14 protein expression in normal donor hearts and failing hearts from patients with dilated cardiomyopathy (*n* = 4 per group, *P < 0.05 vs. normal donor heart). **b** Western blot analysis of ANP, β-MHC, and RGS14 protein expression in hypertrophic hearts from experimental mice undergoing AB (*n* = 4 mice per group, *P < 0.05 vs. sham). **c** Western blot analysis of ANP, β-MHC, and RGS14 in cultured neonatal rat cardiomyocytes stimulated by Angiotensin II (1 μmol/L; *n* = 4) for 24 or 48 h (*P < 0.05 vs. PBS). *Left* Representative western blot. *Right* Bar graphs: quantitative results. *n* indicates the number of independent experiments. The data are presented as the mean ± SD

To investigate if the expression of RGS14 was altered during the process of pathological remodelling, we first measured RGS14 expression in human failing hearts, murine hypertrophic hearts, and NRCMs. According to the western blotting results, the RGS14 protein levels in human failing hearts were reduced to approximately 40 % of the levels in normal donor hearts. This decrease was accompanied by an enhancement of the foetal gene profile of ANP and β-MHC (Fig. 1a). Similar results were found in murine hypertrophic hearts and NRCMs. As shown in Fig. 1b, the expression levels of RGS14 were approximately two-fold and four-fold lower in the experimental mouse hearts after 4 or 8 weeks of AB, respectively, compared with the sham-operated control group. In addition, the expression levels of ANP and β-MHC were dramatically increased at week 4 and more pronounced at week 8 (Fig. 1b). Furthermore, stimulation of NRCMs with Ang II (1 μmol/L) for 24 or 48 h led to RGS14 downregulation and β-MHC and ANP up-regulation (Fig. 1c). The expression of RGS14 was not significantly changed in response to AngII stimulation for 24 h in the isolated cardiac fibroblasts (Figure S1). These results indicated that RGS14 in cardiomyocytes was markedly altered by hypertrophic stress in vivo and in vitro, which demonstrated that RGS14 might be involved in cardiac remodelling.

### RGS14 protects against angiotensin II-induced cardiomyocyte hypertrophy in vitro

**Fig. 2:**
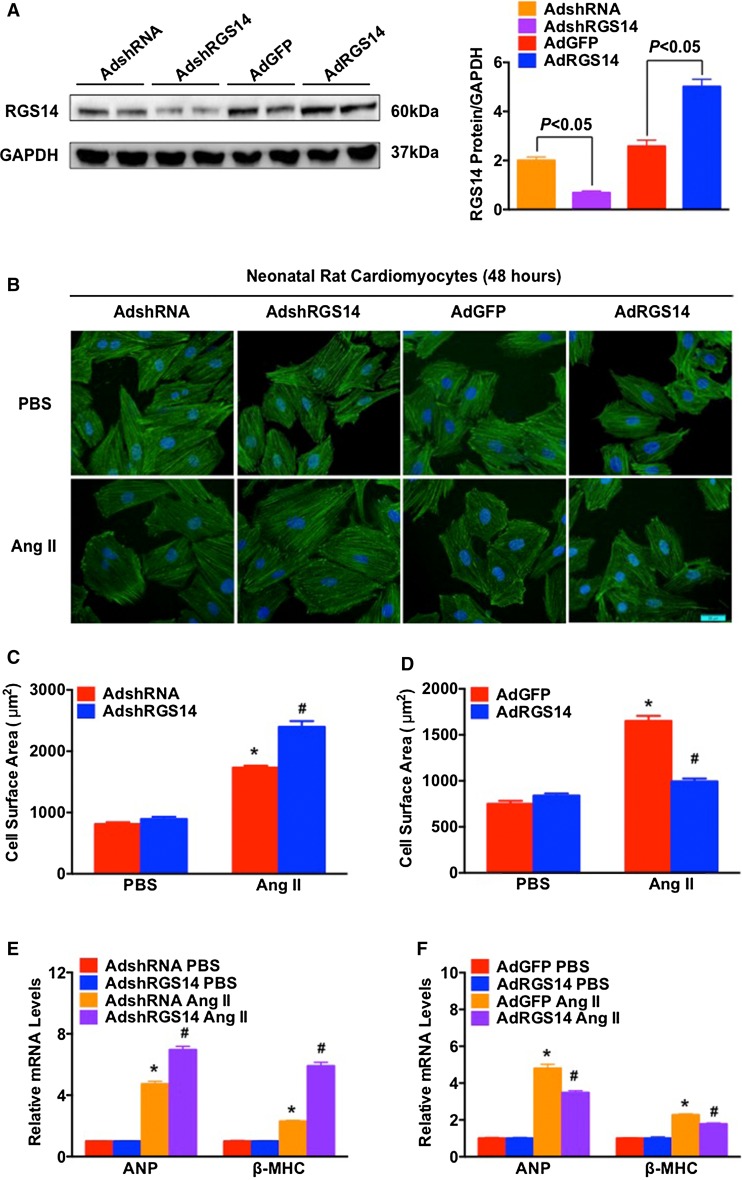
RGS14 protects against Ang II-induced cardiomyocyte hypertrophy in vitro. **a** RGS14 protein expression in NRCMs infected with AdRGS14, AdshRGS14, or respective controls (AdGFP or AdshRNA). **b** Representative anti-α-actin antibody staining images of NRCMs infected with AdRGS14, AdshRGS14, or respective controls in response to PBS and Ang II (1 μmol/L) treatment for 48 h (*blue* nucleus, *green* α-actinin, *scale bar* 20 μm). **c** Quantitative results of the CSA of NRCMs infected with AdshRGS14 compared with AdshRNA in response to PBS and Ang II (*n* > 40 cells per group, *P < 0.05 vs. AdshRNA/PBS; ^#^ *P* < 0.05 vs. AdshRNA/Ang II). **d** Quantitative results of the CSA of NRCMs infected with AdRGS14 compared with AdGFP in response to PBS and Ang II (*n* > 40 cells per group, *P < 0.05 vs. AdGFP/PBS; ^*#*^ *P* < 0.05 vs. AdGFP/Ang II). **e** Real-time PCR evaluation of the mRNA levels of ANP and BNP in PBS- and Ang II-treated NRCMs infected with AdshRGS14 or AdshRNA (*n* = 4, *P < 0.05 vs. AdshRNA/PBS; ^#^ *P* < 0.05 vs. AdshRNA/Ang II). **f** Real-time PCR evaluation of the mRNA levels of ANP and β-MHC in PBS- and Ang II-treated NRCMs infected with AdRGS14 or AdGFP (*n* = 4, *P < 0.05 vs. AdGFP/PBS; ^#^ *P* < 0.05 vs. AdGFP/Ang II). The data are presented as the mean ± SD

To explore the functional contribution of RGS14 to the hypertrophy of cardiomyocytes in vitro, we first performed studies using primary cultured NRCMs infected with AdshRGS14, AdRGS14, or control vectors, and further stimulated with Ang II (1 μmol/L) for 48 h. The protein expression of RGS14 is shown in Fig. 2a and S2A. Immunostaining with the α-actin antibody suggested that neither the NRCMs infected with AdRGS14 nor the NRCMs infected with AdshRGS14 exhibited altered cell size compared with the NRCMs infected with AdGFP or AdshRNA under basal conditions. However, AdshRGS14 treatment enhanced the Ang II-induced increase in cell size (Fig. 2b, c). Furthermore, the mRNA levels of ANP and β-MHC were approximately 1.5- and 2.4-fold higher, respectively, in the NRCMs infected with AdshRGS14 after exposure to Ang II than in the controls (Fig. 2e). The cells with overexpression of RGS14 (AdRGS14) exhibited significantly reduced cell-surface areas (approximately 40 %) compared with the AdGFP-infected cells, and the mRNA expression of ANP and β-MHC was significantly decreased (approximately 25 %) in AdRGS14-infected cells compared with the cells infected with AdGFP (Fig. 2b, d, f). Together, these observations indicate that RGS14 protected against the hypertrophic response in cardiomyocytes.

### Ablation of *RGS14* exacerbates pressure overload-induced remodelling

**Fig. 3:**
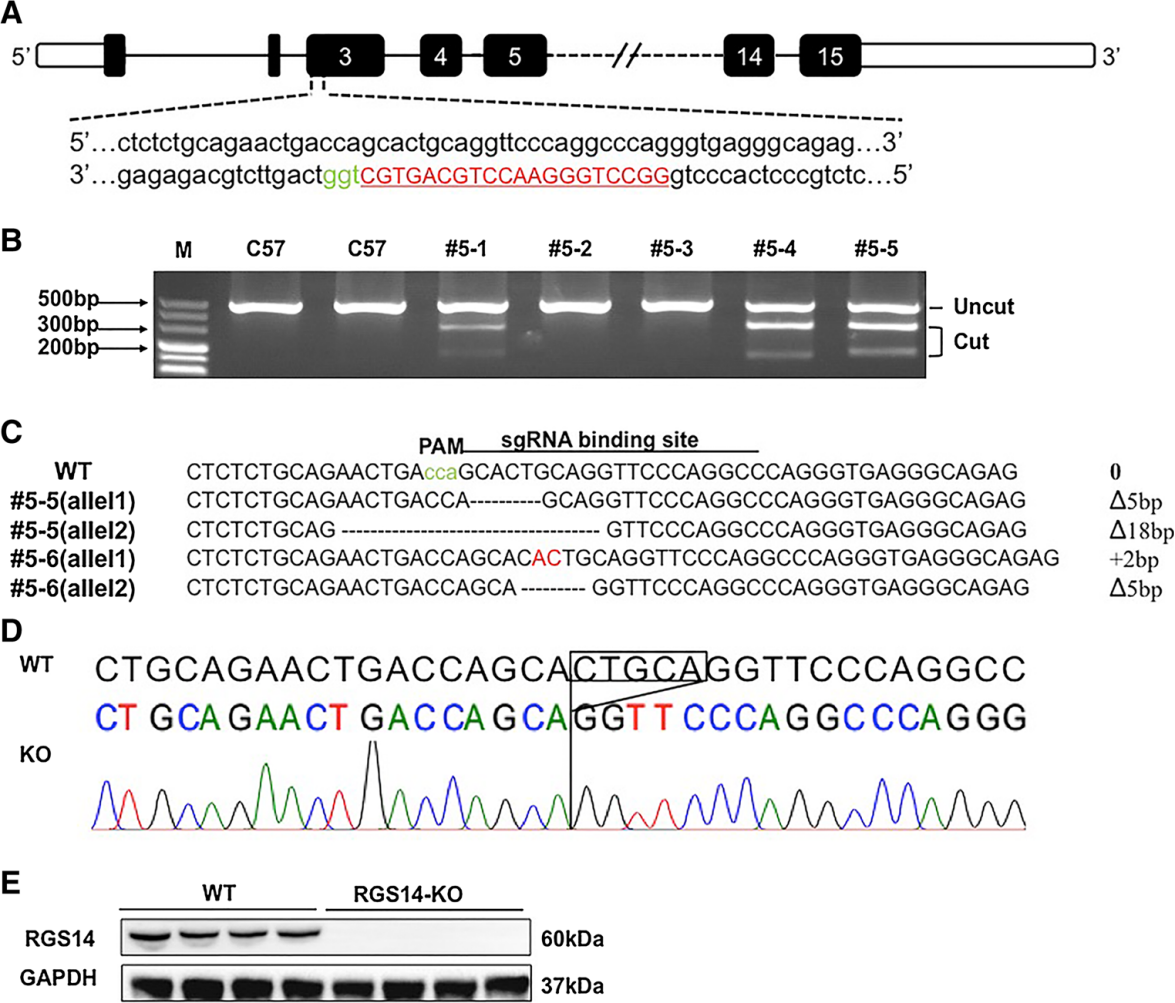
Schematic diagram of the construction of *RGS14*-KO mice using the CRISPR-Cas9 method and identification of RGS14 expression. **a** One sgRNA targeting a region downstream of the 3′ end of exon 3 in the *RGS14* mouse gene was designed and constructed. **b** After microinjection, a T7E1 assay indicated that four out of six pups contained cleavage products, suggesting a mixture of mutant and wild-type DNA templates in these mice. **c** Following subcloning of the PCR products, eight subclones of each mouse were sequenced. All of the subclones carried a single mutant allele, whereas two indels (#5–5, #5–6) produced frameshift mutations. Founder #5–5 was mated to a C57BL/6J mouse to obtain the F1 generation. **d** Gene sequencing for RGS14 expression levels in hearts from wild-type and *RGS14* knockout groups. **e** Representative western blots for RGS14 expression levels in hearts from the *RGS14* ^+/+^ and *RGS14* ^−/−^ groups

**Fig. 4:**
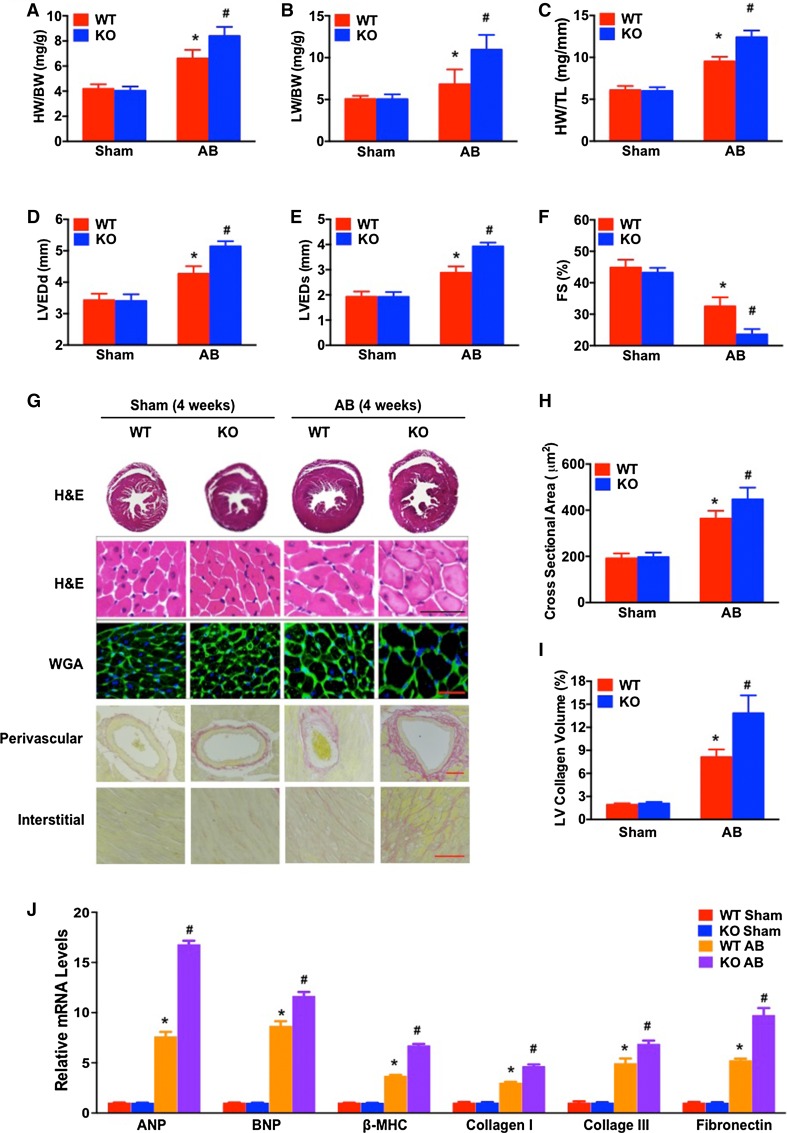
*RGS14* ablation exacerbates pressure overload-induced cardiac hypertrophy. **a**–**c** The HW/BW, LW/BW, and HW/TL ratios were measured in *RGS14* ^+/+^ and *RGS14* ^−/−^ mice 4 weeks after sham or AB treatment, *n* = 12–13 for each group. **d**–**f** Echocardiographic parameters (LVEDd, LVESd, and FS%) for *RGS14* ^+/+^ or *RGS14* ^−/−^ mice after sham treatment and AB treatment (*n* = 4–7 per group). **g** Sections of hearts from *RGS14* ^+/+^ and *RGS14* ^−/−^ mice subjected to AB or sham treatment were stained with H&E (*first row*: *scale bar* 50 μm), WGA (*second row*: *scale bar* 50 μm), and PSR (*third* *row* and *fourth row*: *scale bars* 50 μm) to analyze cardiac hypertrophy and fibrosis (*n* = 5 per group). **h** Quantification of cardiomyocyte cross-sectional area in sham-treated and AB-treated *RGS14* ^+/+^ or *RGS14* ^−/−^ mice (*n* = 5 per group). **i** Quantification of fibrosis areas in sham-treated and AB-treated *RGS14* ^+/+^ or *RGS14* ^−/−^ mice (*n* = 5 per group). **j** mRNA levels of hypertrophic and fibrotic markers in the hearts of *RGS14* ^+/+^ and *RGS14* ^−/−^ mice subjected to sham treatment or AB treatment (*n* = 4 per group). The data are presented as the mean ± SD. *P < 0.05 vs. WT/sham. ^#^ *P* < 0.05 vs. WT/AB

The potential function of RGS14 during cardiac remodelling in vivo was investigated. *RGS14*-KO mice were generated using the CRISPR/Cas9 methods (Fig. 3a–c). Gene sequencing and western blotting of RGS14 expression in heart tissue from *RGS14*-KO and littermate control mice were performed (Fig. 3d, e). At baseline, the *RGS14*-KO mice displayed normal cardiac morphology and contractile function (Table S1). The levels of RGS2, 3, 4, and 5 were not significantly changed in the *RGS14*-KO mice compared with the WT mice (Figure S3). As shown in Fig. 4a, aortic banding induced a 58 % increase in the ratio of heart weight to body weight (HW/BW), indicating the development of cardiac hypertrophy in wild-type mice. The *RGS14*^−/−^ mice exhibited a significantly aggravated hypertrophic effect with an increase of approximately 27 % in the ratio of HW/BW compared with the WT mice subjected to AB surgery. Similar effects were observed in the ratio of lung weight to body weight (LW/BW) and in the ratio of heart weight to tibia length (HW/TL) (Fig. 4b, c). No comparable differences were observed in the sham-treated *RGS14*^−/−^ and the WT mice (Fig. 4a–c). Cardiac function was also measured by echocardiography. The parameters of LVEDd, LVESd, and FS% indicated that myocardial contraction in the AB-treated *RGS14*^−/−^ mice was reduced compared with the WT group subjected to AB (Fig. 4d–f). H&E and WGA staining showed a greater ventricular CSA in the *RGS14*^−/−^ mice than in the control mice subjected to AB surgery (Fig. 4g, h). Because fibrosis is a classical feature of pathological cardiac remodelling and is characterized by the accumulation of collagen in the heart [48], we evaluated the effects of *RGS14* deletion on cardiac fibrosis in pressure-overloaded hearts. Fibrosis was determined by visualizing the extent of collagen staining and calculating the total collagen volume. Both perivascular and interstitial fibrosis analyses consistently demonstrated an increased fibrotic response in the AB-treated *RGS14*^−/−^ mice compared with the AB-treated WT mice (Fig. 4g, i). We measured the synthesis of collagen by analyzing the mRNA expression of hypertrophic markers (ANP, BNP, and β-MHC) and fibrotic markers (collagen I, collagen III, and fibronectin) (Fig. 4j). Our results consistently revealed an increased fibrotic response in *RGS14*^−/−^ hearts. Collectively, these findings reveal that ablation of RGS14 exacerbates hypertrophy and fibrosis in response to chronic pressure overload.

### Overexpression of RGS14 attenuates pressure overload-induced cardiac remodelling

**Fig. 5:**
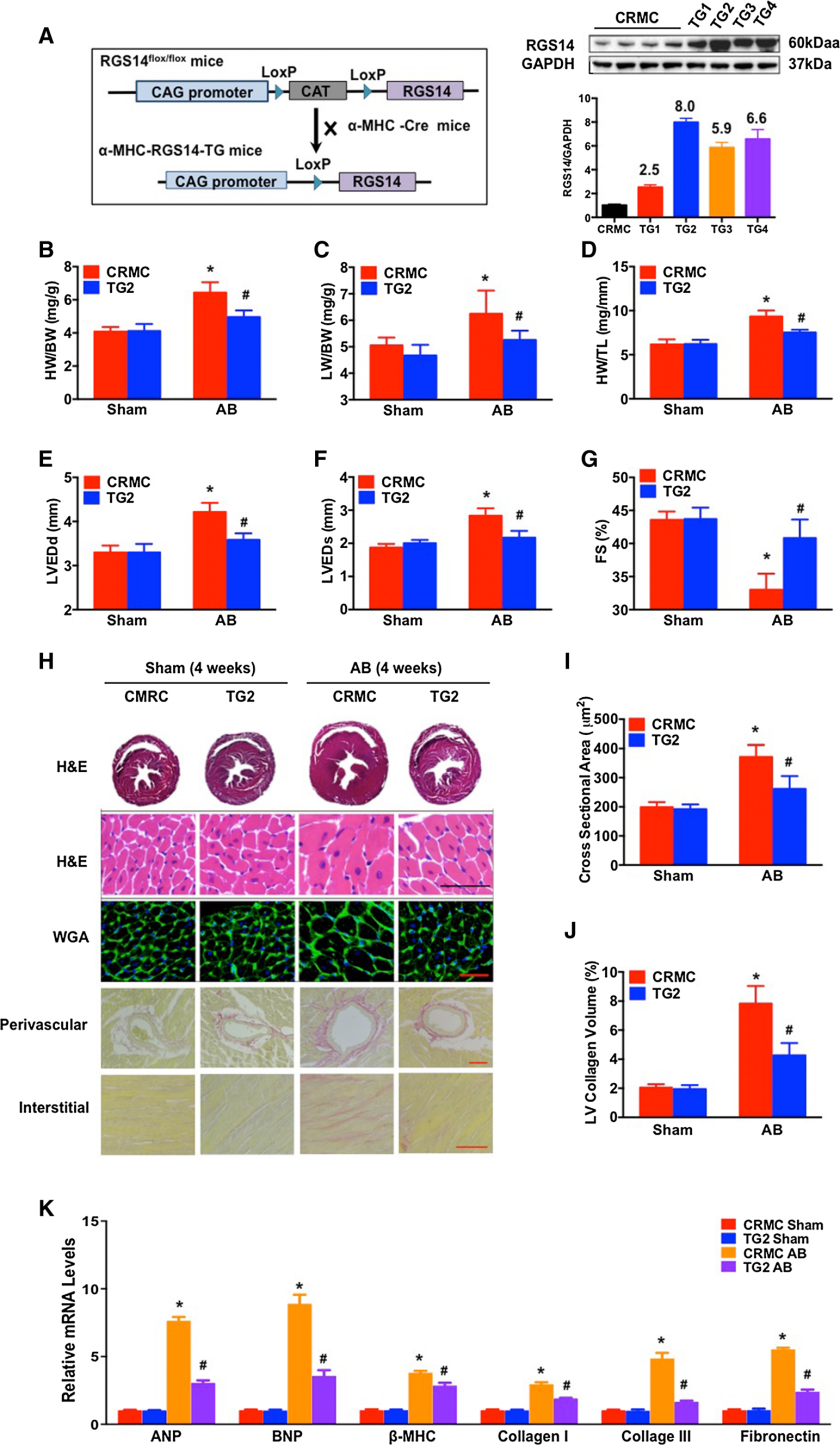
*RGS14*-TG mice are protected from AB-induced cardiac hypertrophy. **a** A schematic diagram of the generation of TG mice with cardiac-specific expression of RGS14 is shown on the *left*. Representative western blots for RGS14 expression levels in hearts from TG and CRMC mice are shown on the *right*. **b**–**d** The HW/BW, LW/BW, and HW/TL ratios in TG2 and CRMC mice after sham treatment or AB treatment for 4 weeks (*n* = 12–13 for each group). **e**–**g** Cardiac function (LVEDd, LVESd and FS) measured by echocardiography for TG2 or CRMC mice after sham treatment and AB treatment (*n* = 6–7 per group). **h** Sections of hearts from TG2 and CRMC mice subjected to AB or sham treatment were stained with H&E (*first row*: *scale bar* 50 μm), WGA (*second row*: *scale bar* 50 μm), and PSR (*third* and *fourth row*: *scale bars* 50 μm) to analyze cardiac hypertrophy and fibrosis (*n* = 5 per group). **i** Quantification of cardiomyocyte CSA in sham-treated and AB-treated TG2 and CRMC mice (*n* = 5 per group). **j** Quantification of fibrosis areas in sham-treated and AB-treated TG2 and CRMC mice (*n* = 5 per group). **k** mRNA levels of hypertrophic and fibrotic markers in the hearts of TG2 and CRMC mice subjected to sham treatment or AB treatment (*n* = 4 per group). The data are presented as the mean ± SD. *P < 0.05 vs. CRMC/sham. ^#^ *P* < 0.05 vs. CRMC/AB

To further confirm the protective effect of RGS14 on cardiac remodelling, cardiac-specific transgenic mice overexpressing murine RGS14 under the control of the CAG promoter were generated, and four independent *RGS14*-TG mice were generated (Fig. 5a). Cardiac RGS14 expression in these TG mice was approximately two- to eight-fold higher than that in their CRMC littermates. TG line 2, carrying the highest levels of RGS14 expression, was selected for further research. At baseline, the *RGS14*-TG2 mice displayed normal cardiac morphology and contractile function (Table S1). The levels of RGS2, 3, 4, and 5 were not significantly changed in the *RGS14*-TG2 mice compared with the CRMC mice (Figure S3). A morphological disparity occurred when comparing *RGS14*-TG2 to CRMC mice 4 weeks after AB. As shown in Fig. 5b, AB induced a 40 % increase in the HW/BW compared with the sham control, suggesting the development of cardiac hypertrophy in the CRMC mice, whereas the TG2 mice exhibited a significant protective effect against hypertrophy, with a decrease of 23 % in the HW/BW. A similar protective effect against hypertrophy was observed in the LW/BW and HW/TL. In contrast to the CRMC mice, the TG2 mice exhibited significant decreases in the LW/BW and HW/TL (Fig. 5c, d). These changes were consistent with the protective role of RGS14 in cardiac function in response to hypertrophic stimulation, as measured by cardiac function parameters (LVEDd, LVESd and FS%) by echocardiography (Fig. 5e–g). Compared with the CRMC mice, the *RGS14*-TG2 mice had smaller heart and cardiomyocyte sizes (Fig. 5h, i) and reduced fibrosis volumes (Fig. 5h, j). Furthermore, AB increased the mRNA levels of ANP, BNP, β-MHC, collagen I, collagen III, and fibronectin in the hearts of the CRMC mice, and these expression levels were significantly suppressed in the *RGS14*-TG2 mice (Fig. 5k). We also examined the hypertrophic response of *RGS14*-TG1 mice (RGS14 expression is 2.5-fold higher than that of CRMC) to evaluate the relevance of RGS14 expression in cardiac hypertrophy. As shown in Figure S4, the heart weight/body weight ratio and the cross-sectional area were slightly, but significantly reduced in the *RGS14*-TG1 group compared with the CRMC controls, 4 weeks after AB surgery. The protective effect of RGS14 on pressure overload-induced cardiac hypertrophy in the *RGS14*-TG1 mice was significantly weaker than in the *RGS14*-TG2 mice, suggesting a possible gene doses effect. Together, these results indicate that overexpression of RGS14 might suppress cardiac remodelling induced by AB in vivo.

### RGS14 suppresses cardiac remodelling via the MEK–ERK1/2 signalling pathway

**Fig. 6:**
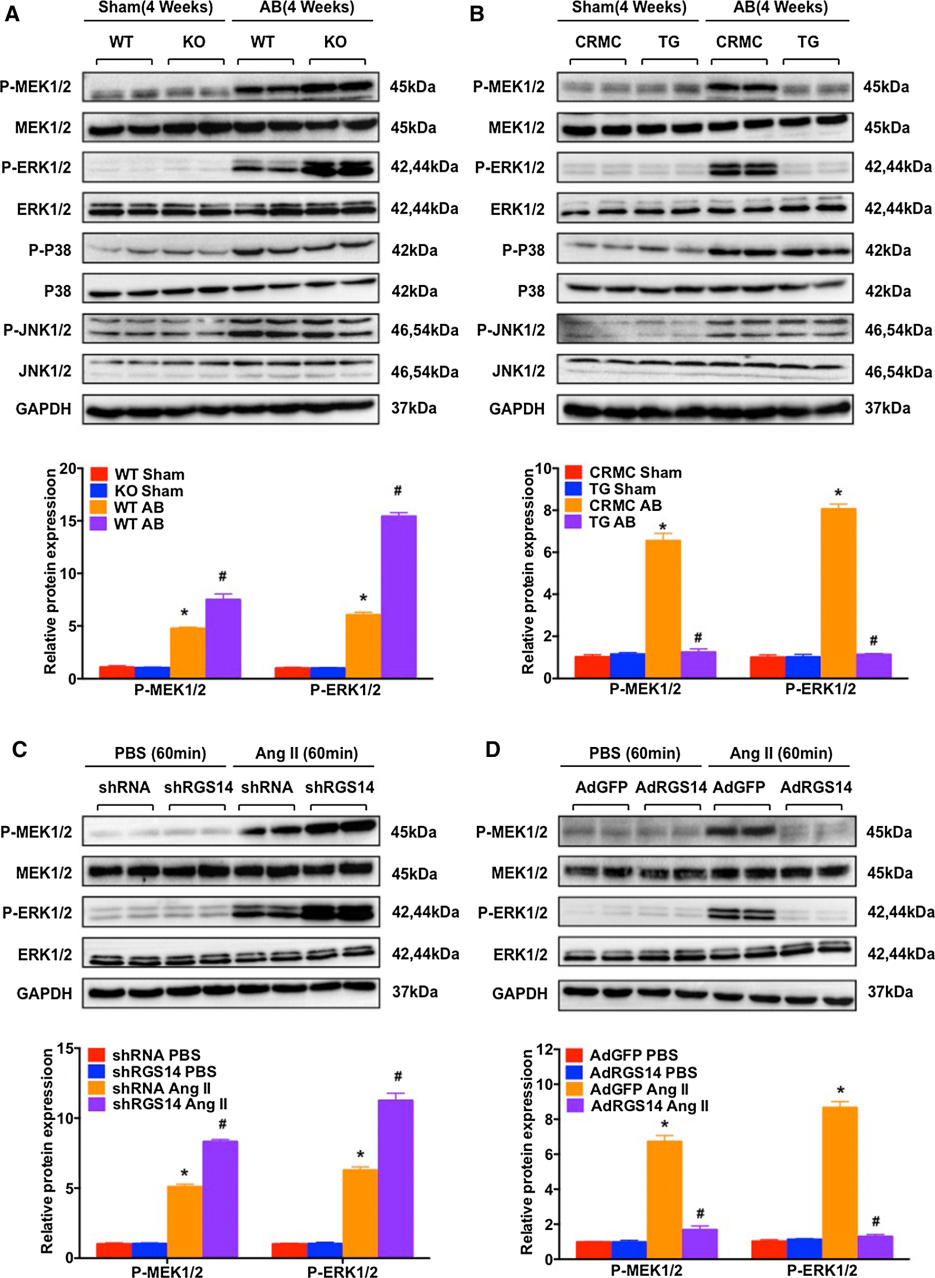
RGS14 inhibits the MEK–ERK1/2 signalling pathway in cardiomyocytes and experimental mice. Representative western blots and quantitative analysis of the phosphorylated and total protein levels of MEK1/2, ERK1/2, JNK1/2, and p38 after sham treatment or AB treatment in WT and *RGS14* ^−/−^ mice (*n* = 4 mice per group; *P < 0.05 vs. WT/sham; ^#^ *P* < 0.05 vs. WT/AB) (**a**) and in CRMC and *RGS14*-TG mice at week four (*n* = 4 mice per group; *P < 0.05 vs. CRMC/sham; ^#^ *P* < 0.05 vs. CRMC/AB) (**b**). Levels of phosphorylated and total MEK1/2 and ERK1/2 proteins in samples of NRCMs infected with AdshRGS14 (**c**) or AdRGS14 (**d**) and treated with Ang II (*n* = 4, *P < 0.05 vs. AdshRNA/PBS or AdGFP/PBS; ^#^ *P* < 0.05 vs. AdshRNA/Ang II or AdGFP/Ang II). *Upper* Representative blots; *lower* quantitative results. The data are presented as the mean ± SD

To determine the underlying mechanism of the anti-hypertrophy effect of RGS14, the expression and activity of MAPK signalling molecules were detected. *RGS14*-TG2 mice were selected for the current research. As shown in Fig. 6a, b, the phosphorylation levels of MEK1/2, ERK1/2, JNK1/2, and p38 were significantly elevated after AB surgery, whereas the total protein expression remained unchanged. Deletion of *RGS14* further increased the activation of MEK1/2 and ERK1/2 by 1.6- and 2.6-fold, respectively, compared with the WT mouse hearts subjected to AB. Overexpression of RGS14 restored the phosphorylation level of MEK1/2 and ERK1/2 to approximately normal. Neither p38 nor JNK phosphorylation was altered in the AB-induced *RGS14*^−/−^ and *RGS14*-TG hearts.

To exclude potential compensatory mechanisms in vivo, further NRCM experiments were conducted. AdshRGS14 and AdRGS14 were used to knock down or overexpress RGS14 expression in NRCMs, respectively. The protein expression of RGS14 in different groups is shown in Figure S2. The expression and activity of MAPK signalling molecules were detected compared with AdshRNA and AdGFP controls. Western blot analysis showed that RGS14 down-regulation enhanced the expression of phosphorylated MEK1/2 and ERK1/2 compared with AdshRNA-infected cells under Ang II treatment (Fig. 6c), whereas RGS14 up-regulation strongly suppressed the levels of MEK1/2 and ERK1/2 phosphorylation in AdRGS14-infected NRCMs compared with the AdGFP-infected group (Fig. 6d). Our results demonstrate that the RGS14-elicited anti-hypertrophic effect is largely associated with the inhibition of MEK–ERK1/2 signalling in hearts.

**Fig. 7:**
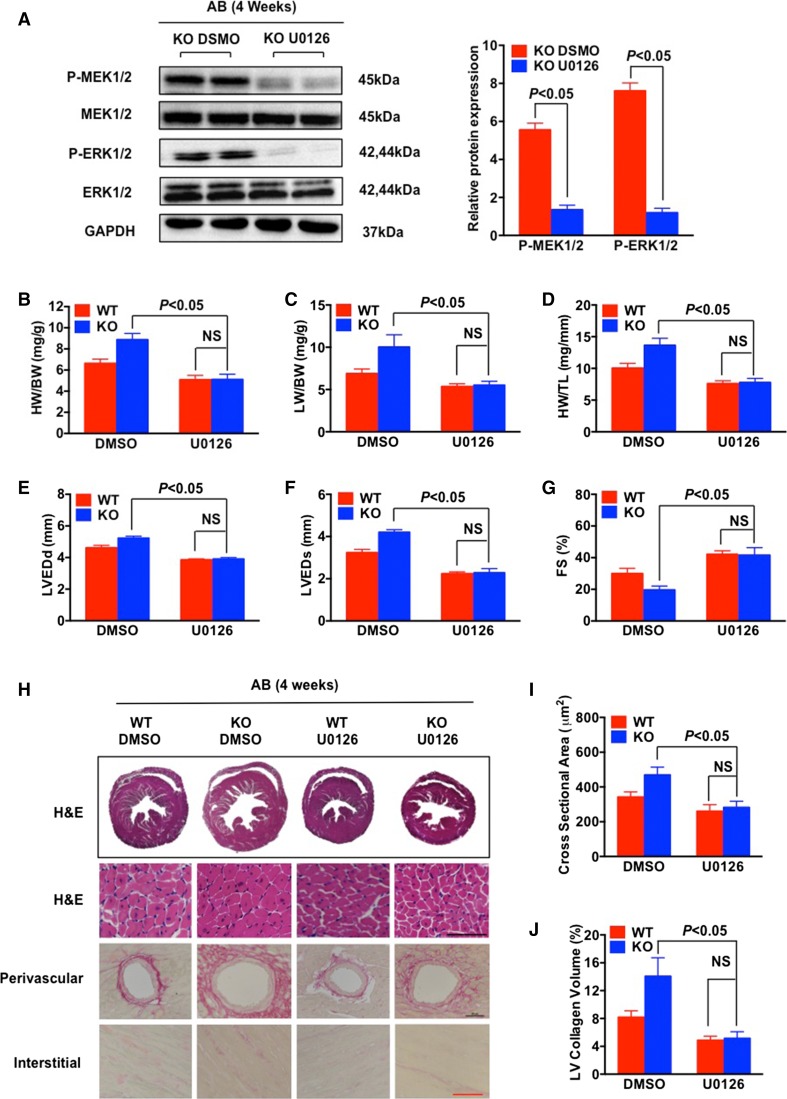
Inhibition of MEK1/2 abolishes cardiac abnormalities in *RGS14* ^−/−^ mice in response to pressure overload. **a** Representative western blotting and quantitative analysis of the phosphorylation levels of MEK1/2 and ERK1/2 in *RGS14* ^−/−^ mice treated with an inhibitor of MEK (U0126) compared with DMSO in response to AB treatment (*n* = 4 mice per group). **b**–**d** The HW/BW, LW/BW, and HW/TL ratios in *RGS14* ^+/+^ and *RGS14* ^−/−^ mice treated with U0126 or DMSO 4 weeks after AB surgery. (*n* = 9 for each group). **e**–**g** Echocardiographic parameters (LVEDd, LVESd, and FS%) for *RGS14* ^+/+^ and *RGS14* ^−/−^ mice treated with U0126 or DMSO after AB surgery (*n* = 8–9 per group). **h** Sections of hearts from *RGS14* ^+/+^ and *RGS14* ^−/−^ mice treated with U0126 or DMSO subjected to AB surgery were stained with H&E (*first row*: *scale bar* 50 μm) and PSR (*second* *row* and *third row*: *scale bars* 50 μm) to analyze cardiac hypertrophy and fibrosis (*n* = 5 per group). **i** Quantification of cardiomyocyte cross-sectional area in *RGS14* ^+/+^ and *RGS14* ^−/−^ mice treated with U0126 or DMSO after AB surgery (*n* = 5 per group). **j** Quantification of fibrosis areas in *RGS14* ^+/+^ and *RGS14* ^−/−^ mice treated with U0126 or DMSO after AB surgery (*n* = 5 per group). *NS* no significance. The data are presented as the mean ± SD

To determine if the MEK–ERK1/2 pathway plays an essential role in RGS14-induced protection in AB-induced cardiac hypertrophy, U0126 (an inhibitor of MEK) was infused into WT and *RGS14*-KO mice before AB surgery. Western blot analysis indicated lower levels of phosphorylated MEK1/2 and ERK1/2 in U0126-treated *RGS14*-KO mice compared with control mice (Fig. 7a). As shown in Fig. 7b–j, U0126 significantly restricted the deteriorative cardiac remodelling in *RGS14*-KO mice in response to AB. In U0126-treated *RGS14*-KO mice, the HW/BW, LW/BW, and HW/TL ratios were decreased compared with the DMSO-control group (Fig. 7b–d), and heart function according to LVEDd, LVESd, and FS% values was improved compared with the DMSO-control group (Fig. 7e–g). Moreover, smaller cross-sectional areas of cardiomyocytes (Fig. 7h, i) and lower collagen volumes (Fig. 7h, j) were observed in the *RGS14*-KO mice treated with U0126 compared with the DMSO-treated controls. These parameters were equal in both the U0126-treated *RGS14*-KO mice and the WT mice (Fig. 7b–j). These findings suggest that pre-inhibition of MEK–ERK1/2 signalling protects against AB-induced cardiac remodelling in *RGS14*-KO mice.

**Fig. 8:**
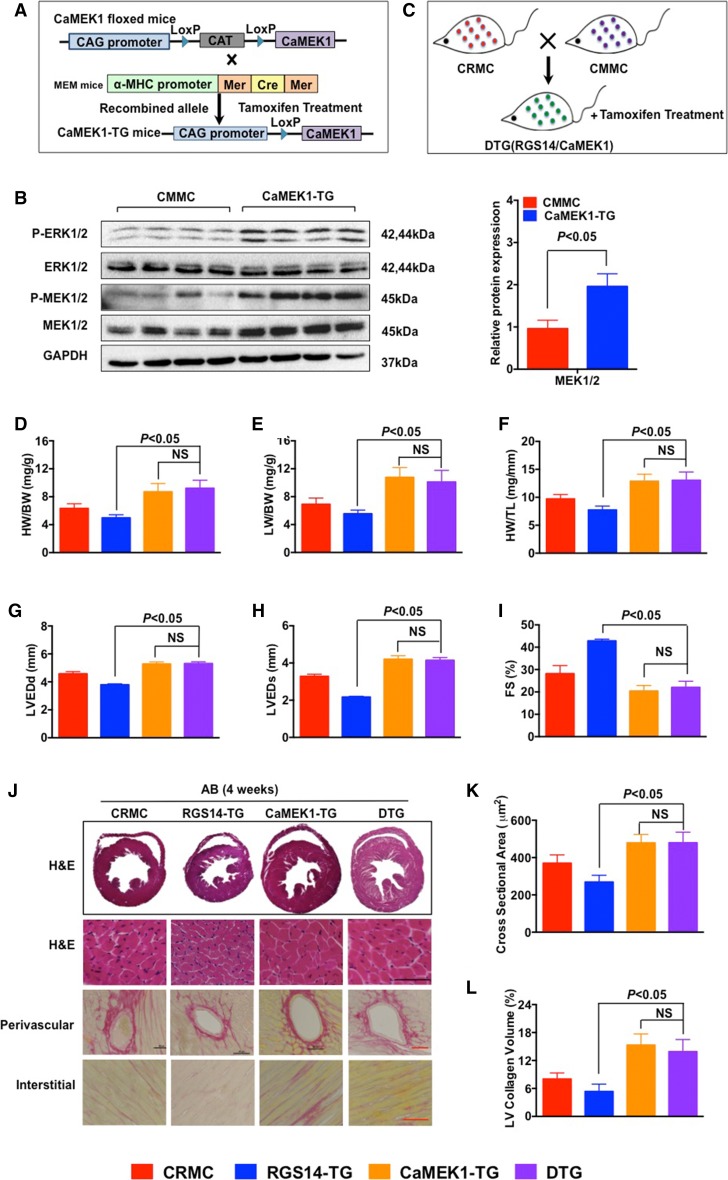
Overexpression of MEK1 negates the protective effects of RGS14 on cardiac hypertrophy in *RGS14*-TG mice. **a** A schematic diagram of the generation of TG mice with cardiac-specific expression of MEK1. **b** Representative western blots of CMMC and *CaMEK1*-TG mice 4 weeks after AB surgery (*n* = 4 per group). The level of MEK was significantly increased in the *CaMEK1*-TG mice compared with the control group. **c** Breeding strategy for the production of *CaMEK1*/*RGS14* double transgenic mice. **d**–**f** The HW/BW, LW/BW, and HW/TL ratios in CRMC, *RGS14*-TG, *CaMEK1*-TG, and DTG mice subjected to AB surgery (*n* = 8–9 for each group), respectively. **g**–**i** Cardiac function (LVEDd, LVESd, and FS%) as measured by echocardiography of CRMC, *RGS14*-TG, *CaMEK1*-TG, and DTG mice after AB treatment (*n* = 6–8 per group). **j** Sections of hearts from CRMC, *RGS14*-TG, *CaMEK1*-TG, and DTG mice subjected to AB surgery were stained with H&E (*first row*: *scale bar* 50 μm) and PSR (*second* *row* and *third row*: *scale bars* 50 μm) to analyze cardiac hypertrophy and fibrosis (*n* = 5 per group). **k** Quantification of cardiomyocyte cross-sectional area in AB-treated CRMC, *RGS14*-TG, *CaMEK1*-TG, and DTG mice (*n* = 5 per group). **l** Quantification of the fibrosis areas in AB-treated CRMC, *RGS14*-TG, *CaMEK1*-TG, and DTG mice (*n* = 5 per group). *NS* no significance. The data are presented as the mean ± SD

Transgenic mice with floxed *CaMEK1* were crossed with transgenic *αMHC*-*MerCre*-*Mer* mice to generate cardiac-specific *CaMEK1* transgenic mice (*CaMEK1*-TG), and transgenes were identified by the western blot analysis (Fig. 8a, b). The level of MEK was significantly increased in the *CaMEK1*-TG mice compared with the CMMC group. *RGS14*/*CaMEK1* DTG mice were generated by crossing CRMC mice with CMMC mice and treating the mice with tamoxifen (Fig. 8c). At baseline, the *CaMEK1*-TG and DTG mice displayed normal cardiac morphology and contractile function (Table S1). The RGS14 and phospho-ERK levels were determined in the *CaMEK1/RGS14* DTG mice, as shown in Figure S5. As expected, the overexpression of CaMEK1 produced more AB-induced cardiac remodelling compared with the CRMC group (Fig. 8d–l). Four weeks after AB surgery, the HW/BW, LW/BW, and HW/TL ratios were significantly increased in the DTG group compared with the *RGS14*-TG group (Fig. 8d–f). In addition, the LVEDd, LVESd, and FS% values according to the echocardiograph indicated deteriorated cardiac function in the DTG group compared with the *RGS14*-TG group (Fig. 8g–i). Moreover, the heart areas, cardiomyocyte cross-sectional areas, and cardiac fibrosis volumes were significantly up-regulated in the DTG mice, as shown in Fig. 8j–l, compared with the *RGS14*-TG mice. These parameters were equal in the DTG mice and the *CaMEK1* mice (Fig. 8d–l). Our results demonstrate that targeted MEK1 activation abolishes the protective effects of RGS14 on cardiac remodelling after AB surgery. Therefore, our findings indicate that the protective role of RGS14 in pathological cardiac remodelling is at least in part due to the inhibition of MEK1 signalling.

## Discussion

We performed an exploratory study to determine the role of RGS14 in cardiac remodelling and its underlying mechanism by gain-of-function and loss-of-function approaches. Our major findings demonstrated that the disruption of RGS14 resulted in an exaggerated pathological cardiac remodelling response, whereas the overexpression of RGS14 alleviated the cardiac hypertrophy and dysfunction induced by aortic banding operation. Furthermore, the results supported that RGS14-mediated cardio-protection was at least partly attributed to inhibition of the MEK–ERK1/2 signalling pathway. For the first time, our results demonstrated a critical role of RGS14 in the pathophysiology process of cardiac remodelling and heart failure.

RGS proteins are believed to reduce the duration and power of GPCRs’ effects and, therefore, participate in pathophysiology processes [13, 54]. Previous studies have demonstrated that RGS14 is expressed in the heart, although its function in the cardiovascular system remains unknown [25, 55, 68]. We first observed that the protein level of RGS14 was decreased in the hearts of DCM patients, which suggested that RGS14 might be involved in the process of cardiac hypertrophy. Because biomechanical stress and neurohumoral factors are major triggers of cardiac hypertrophy, aortic banding and angiotensin II were used to treat animal models and NRCMs, respectively. The results showed that RGS14 was significantly decreased after aortic banding or angiotensin II stimulation. Furthermore, *RGS14* knockout aggravated cardiac hypertrophy after aortic banding, and RGS14 cardiomyocyte-specific overexpression significantly alleviated cardiac remodelling in vivo, which revealed a protective role of RGS14 in cardiac remodelling.

Molecular mechanism research revealed that MAPK signalling mediated the effect of RGS14 on cardiac hypertrophy. The MAPK cascade comprises a sequence of successive kinases, including p38, JNKs, and ERKs [18, 39, 45]. All three major MAPK pathways are activated in cardiac tissue in pressure overload-induced animal models and in humans with heart failure [14, 16]. It has been reported that JNK is an important mediator of pathological cardiac hypertrophy, although in the animal model with a loss of functional MEK4 (up-stream of JNK), JNK shows controversial effect on cardiac remodelling [10, 35]. P38 plays an essential role in fibrosis, apoptosis, inflammation, and the production of cytokines, but the existing data concerning the role of p38 in hypertrophy in the heart are difficult to reconcile [1]. We found that the activation of MEK–ERK1/2 was inhibited by cardiac RGS14 overexpression, whereas the deletion of RGS14 further enhanced the activation of MEK–ERK1/2 after chronic pressure overload. However, RGS14 did not affect the phosphorylation of p38 and JNK1/2, which indicated that ERK1/2 was the sole downstream target of RGS14 in cardiac remodelling. Furthermore, U0126 mitigated the aggravated effects of RGS14 deficiency on cardiac remodelling, whereas targeted MEK1 activation negated the protective effects of RGS14 on cardiac remodelling. Taken together, mechanistic inhibition of MEK–ERK1/2 signalling could largely account for the cardio-protective effect of RGS14 on pathological cardiac remodelling in the current study.

It is well accepted that the MEK–ERK1/2 signalling pathways are central mediators of cardiac hypertrophy [16, 18, 27, 38, 42]. In the present study, the inhibition of MEK reversed the poor outcomes of cardiac hypertrophy, fibrosis, and dysfunction, whereas the overexpression of MEK1 in *CaMEK1* transgenic mice promoted cardiac hypertrophy. These results suggested a promoting role of MEK–ERK1/2 in pressure overload-induced remodelling. There are reports demonstrating that activated MEK–ERK1/2 signalling resulted in concentric hypertrophy in MEK1 transgenic mice. Mice lacking ERK1/2 in the heart by a genetic approach showed eccentric cardiac growth with and without the AngII stimulation [4, 5, 27]. Therefore, it appeared that MEK–ERK1/2 induced a compensatory mechanism from eccentric to concentric status in cardiac hypertrophy. The effectiveness and specificity of the pharmacological inhibitory and loss-of-function approach in ERK might account for this difference. Furthermore, MEK–ERK might play different roles in cardiac remodelling when receiving different stimuli. Experiments on baseline activation, post-stimulus peak activation, or activation amplitude of MEK–ERK would provide new insight into the role of MEK–ERK pathway in cardiac function.

How RGS14 exhibits an inhibitory effect on the MEK1/2-ERK1/2 cascade in cardiac remodelling remains unclear. In addition to the conserved RGS domain, RGS14 contains the GoLoco domain and two Ras/Rap-binding domains [9, 57, 58, 69]. The RGS domain and GoLoco motif proteins in RGS14 are referred to bind to Gi and inhibit its guanine nucleotide dissociation [58]. Gi is best described as the inhibitory isoform of Gα that suppresses adenylate cyclase activity, leading to decreased cAMP accumulation [2, 63]; however, to our knowledge, there are no data demonstrating that the loss of Gi regulates cardiac remodelling. Several studies have indicated that over-activation of Ras signalling induces pathological cardiac remodelling through the MER-ERK cascade pathway in vivo and in vitro [17, 20, 42]. In addition, the Ras-binding domain was defined as the binding site of RGS14 when regulating the MAPK signalling pathway in a synaptic plasticity study [61]. Therefore, it is possible that the Ras-binding domain of RGS14 is responsible for the inhibitory effect of RGS14 on the MEK–ERK1/2 cascade in cardiac remodelling. The specific mechanism distinguishes RGS14 as the special protein among the RGS proteins in cardiac remodelling, although the further implication needs more exploration.

RGS2, 3, 4, and 5, which belong to the R4/B subfamily, have been demonstrated to play a protective role in pressure overload-induced cardiac remodelling [31, 36, 47, 52, 53, 62]. In the present study, the levels of RGS 2, 3, 4, and 5 were not changed in the *RGS14*-KO mice compared with the wild-type mice or in the *RGS14*-TG mice compared with the CRMC mice. Therefore, it appeared that there was no complementary mechanism between RGS14 and other RGS proteins in cardiac remodelling.

RGS14 was expressed both in cardiomyocytes and cardiac fibroblasts, although the level of RGS14 was unchanged in fibroblasts in response to angiotensin II stimuli in the current study, suggesting that profibrotic signalling in fibroblasts might not be linked directly to RGS14 in pathological processes. RGS14 overexpression only in cardiomyocytes appeared to be sufficient to protect against pressure overload-induced cardiac remodelling. Previous studies have indicated that activated Ras-MEK–ERK1/2 from cardiomyocytes could markedly reduce fibrosis in response to pressure overload [60, 67], which might explain the underlying mechanism of RGS14 on cardiac fibrosis. We are unable to exclude the possibility that RGS14 in fibroblasts might contribute to cardiac hypertrophy via other pathways.

A limitation of this study is that the up-stream regulatory mechanism for RGS14-mediated protection of heart hypertrophy was not elucidated, because we only focused on the effect of RGS14 on the development of heart remodelling in this study. Numerous reports have indicated that RGS could be regulated by a variety of factors, including GPCR activation, second messengers, and epigenetic changes in different cell types [46, 59]. RGS appeared to be a common downstream mediator in heart remodelling. The present study suggested that RGS14 could respond to pressure overload and Ang II, but more details should be studied in future.

Our research demonstrated that RGS14 protected the development of cardiac hypertrophy via suppressing the MEK–ERK1/2 signalling pathway in vitro and in vivo. These observations implied that RGS14 is a newly appreciated partner of GPCRs in the heart. RGS proteins could serve as potential therapeutic targets for cardiac hypertrophy and heart failure.

## Electronic supplementary material

Below is the link to the electronic supplementary material.

Supplementary material 1 (DOCX 2934 kb)
