# Supplementary material for: Regulator of G protein signalling 14 attenuates cardiac remodelling through the MEK–ERK1/2 signalling pathway
Source: Basic Res Cardiol. 2016 Jun 13;111:47. doi: 10.1007/s00395-016-0566-1 (PMC4906057; doi:10.1007/s00395-016-0566-1)
Supplement: Supplementary file 1 — Supplementary material 1 (DOCX 2934 kb) [file 395_2016_566_MOESM1_ESM.docx]

**Supplemental Table**

| **Parameter** | **WT mice**  **（n=15）** | **RGS14-KO mice**  **（n=14）** | **CRMC**  **（n=13）** | **RGS14-TG**  **（n=12）** | **CaMEK1-TG**  **（n=13）** | **DTG mice**  **（n=11）** |
| --- | --- | --- | --- | --- | --- | --- |
| BW (g) | 25.79±1.19 | 26.55±0.98 | 26.14±1.18 | 25.57±1.30 | 26.29±1.05 | 26.03±0.81 |
| HW/BW(mg/g) | 4.12±0.29 | 4.25±0.33 | 4.18±0.21 | 4.23±0.34 | 4.20±0.32 | 4.27±0.37 |
| LW/BW(mg/g) | 5.26±0.40 | 5.24±0.32 | 5.37±0.31 | 5.49±0.53 | 5.36±0.55 | 5.28±0.51 |
| HW /TL(mg/mm) | 6.14±0.28 | 6.27±0.47 | 6.23±0.44 | 6.31±0.70 | 6.26±0.55 | 6.20±0.52 |
| HR (beats/min) | 518.33±36.34 | 534.43±32.87 | 508.50±39.47 | 524.17±35.78 | 512.69±51.29 | 541.00±44.47 |
| LVEDd (mm) | 3.62±0.13 | 3.65±0.11 | 3.59±0.12 | 3.64±0.08 | 3.60±0.10 | 3.68±0.10 |
| LVESd (mm) | 1.93±0.10 | 2.00±0.10 | 1.95±0.07 | 1.98±0.11 | 2.02±0.08 | 1.96±0.08 |
| FS (%) | 46.87±2.17 | 45.86±2.74 | 45.58±2.35 | 46.17±2.41 | 45.00±1.29 | 47.18±2.32 |

Table S1: ﻿Anatomic and Echocardiographic Analysis in 8-10 Week Old Mice

BW=body weight; HW=heart weight; LW=lung weight; TL=tibial length; HR=heart rate; LVEDd =left ventricular end-diastolic diameter; LVESd =left ventricular end-systolic diameter; FS=fractional shortening, All values are presented as the mean ± SD.

**Supplemental Figures**

**
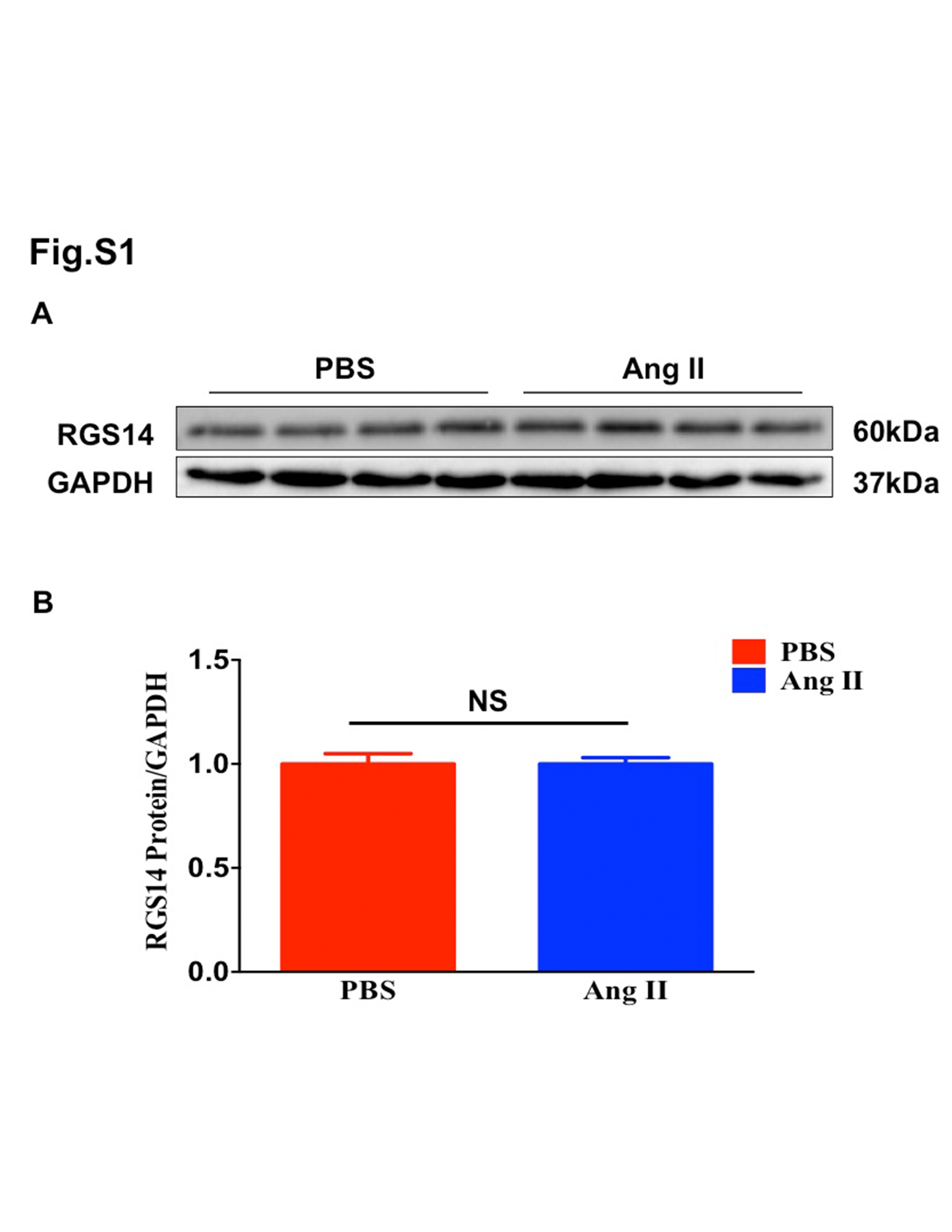
**

**Figure S1. The protein level of RGS14 in neonatal cardiac fibroblasts.** Ang II stimulation for 24 hour did not cause significant changes of RGS14 expression in neonatal cardiac fibroblasts compared to PBS. NS: no significance.


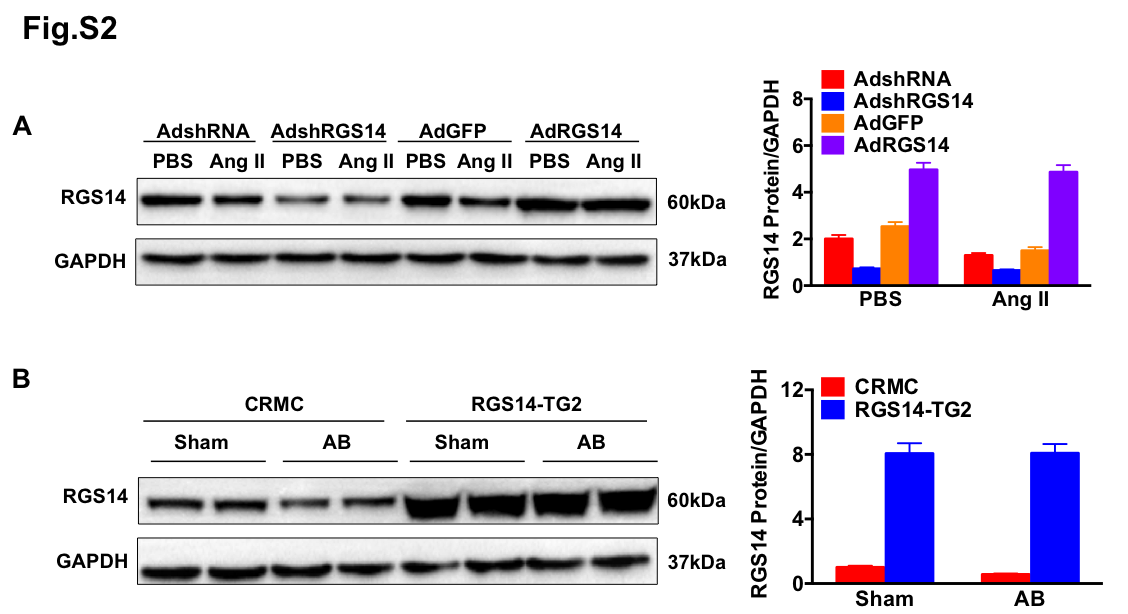


**Figure S2. The expression of RGS14 under different conditions in vitro and in vivo. A,** the RGS14 level under AdshRGS14 and AdRGF14 transfection with or without Ang II stimulation. **B,** the RGS14 level in CRMC and RGS14-TG animals 4 weeks after aortic banding.


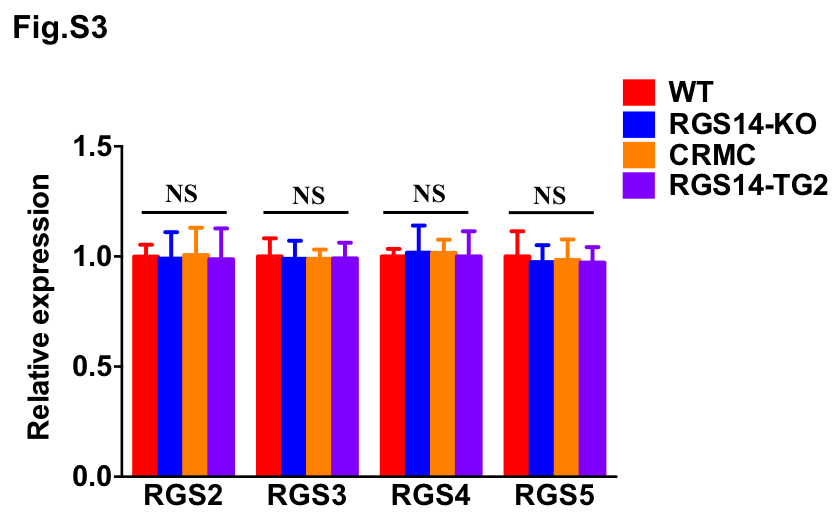


**Figure S3. The level of RGS 2, 3, 4 and 5 in heart samples from RGS14 KO and TG mice.** To investigate the complementary mechanism of RGS proteins, mRNA level of RGS 2, 3, 4 and 5 were determined in heart samples from RGS14 KO and TG mice and no significant changes were found. NS: no significance. (n=10 per mice group)

**
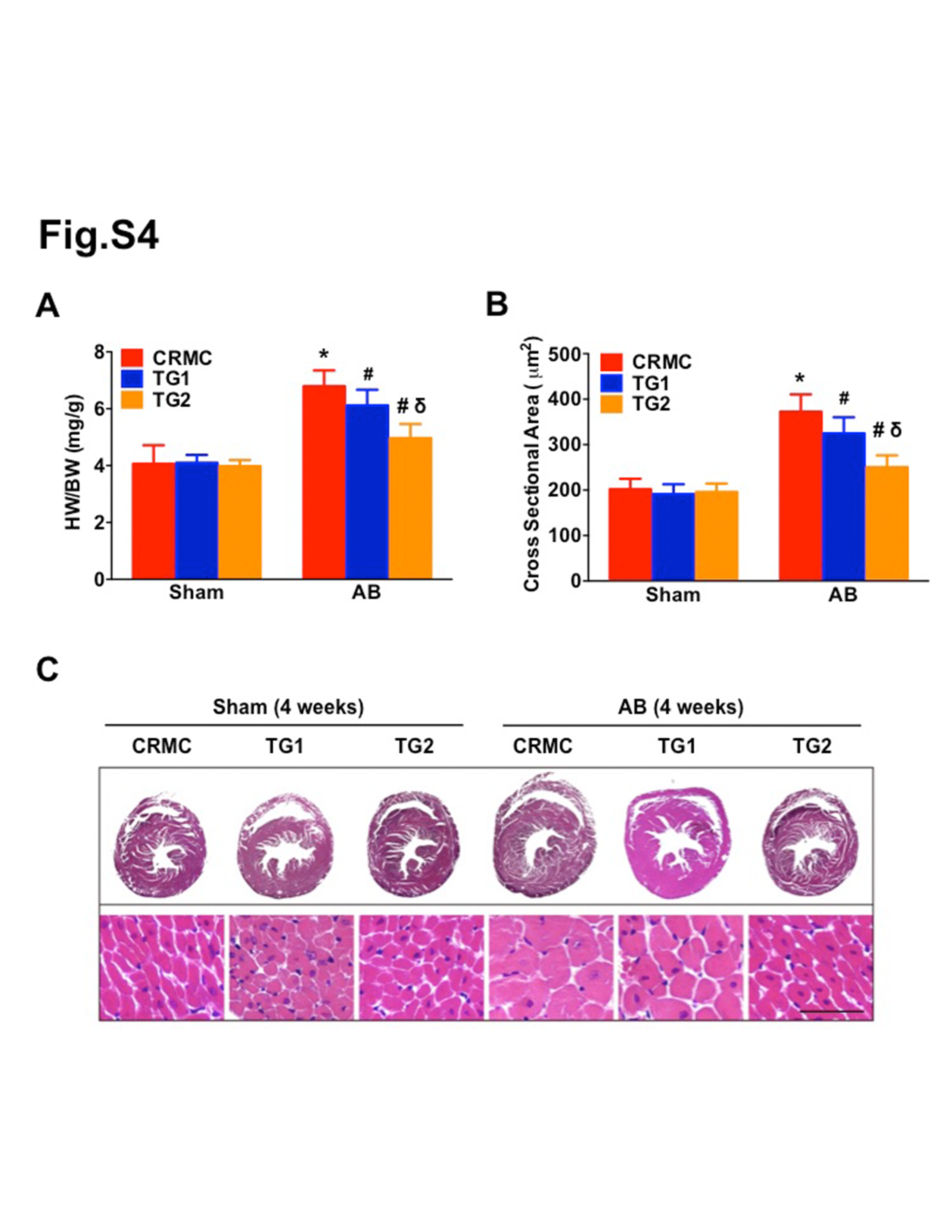
**

**Figure S4. Two lines of RGS14-TG mice are protected from AB-induced cardiac hypertrophy. A,** The HW/BW in TG1,TG2 and CRMC mice 4 weeks after sham treatment or AB treatment (n=12-13 for each group). **B,** Quantification of cardiomyocyte CSA in TG1, TG2 and CRMC mice treated with or without AB (n=5 per group). **C** Sections of hearts from TG and CRMC mice subjected to AB or sham treatment were stained with H&E (scale bars=50 μm), The data are presented as the means ± SD. * *P*＜0.05 vs. CRMC/sham. #*P*＜0.05 vs. CRMC/AB. δ*P*＜0.05 vs. TG1/AB.


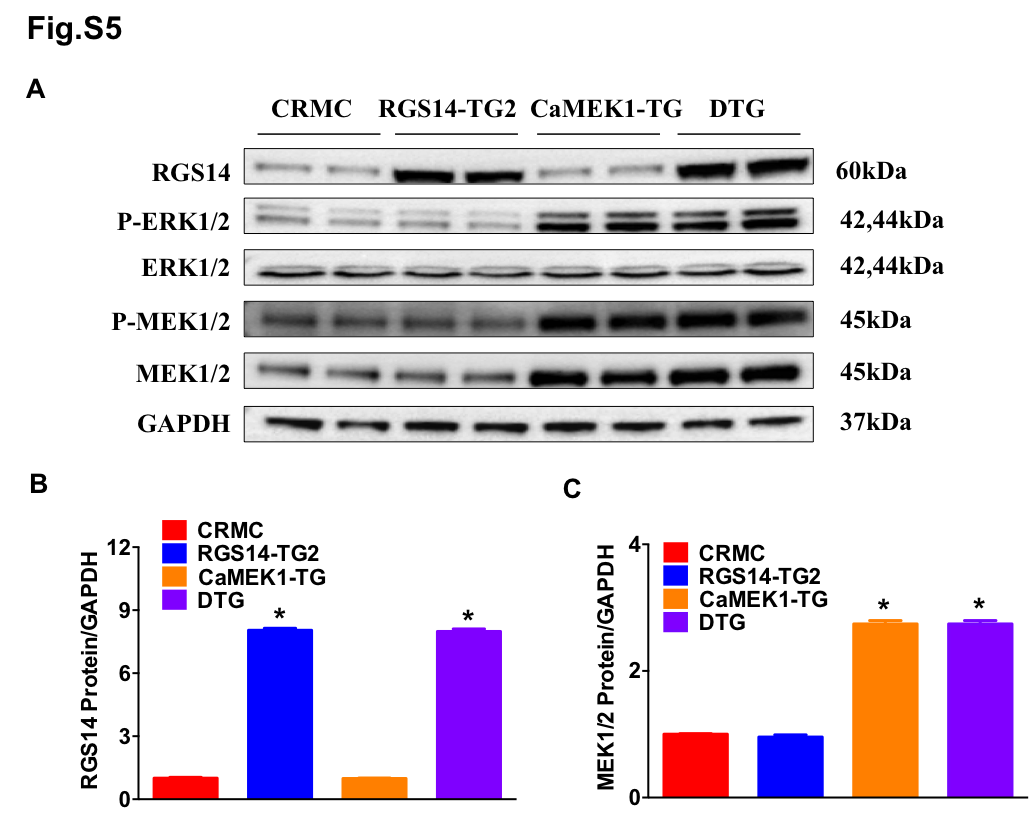


**Figure S5. Protein level of RGS14 and MEK1/2 in RGS14-TG mice, CaMEK1-TG mice and DTG mice. A,** Representative western blots results of RGS14 and MEK1/2 in three transgenic mice (n=11-13 per group). **B**, The quantification of protein level of RGS14 shown in A. **C**, The quantification of protein level of MEK1/2 shown in A.* *P*＜0.05 vs CRMC.
